# Differential Circulating Fungal Microbiome in Prostate Cancer Patients Compared to Healthy Control Individuals

**DOI:** 10.1155/2022/2574964

**Published:** 2022-02-03

**Authors:** Xu Wang, Zejun Zhou, David Turner, Michael Lilly, Tongwen Ou, Wei Jiang

**Affiliations:** ^1^Department of Microbiology and Immunology, Medical University of South Carolina, Charleston, 29425 SC, USA; ^2^Department of Urology, Capital Medical University Affiliated XuanWu Hospital, 45 Changchun Street, Xicheng District, Beijing 100053, China; ^3^State Key Laboratory of Developmental Biology of Freshwater Fish, College of Life Sciences, Hunan Normal University, Changsha 410081, China; ^4^Department of Pathology and Laboratory Medicine, Medical University of South Carolina, Charleston, SC, USA; ^5^Division of Hematology and Oncology, Department of Medicine, Medical University of South Carolina, Charleston, SC 29425, USA; ^6^Division of Infectious Diseases, Department of Medicine, Medical University of South Carolina, Charleston, SC 29425, USA

## Abstract

**Backgrounds:**

Infection and inflammation play an important role in prostate cancer (PCa) etiology and pathogenesis. However, the environmental drivers for PCa are not fully understood.

**Methods:**

In a cross-sectional study, we analyzed circulating fungal microbiome in plasma samples from age and race-matched healthy control men (*n* = 34) and preoperative PCa patients (*n* = 31).

**Results:**

The fungal community in the plasma exhibited differences between individuals with PCa and healthy controls according to the beta diversity; there was no difference in the alpha diversity. Moreover, the relative abundance of several fungi differed between the two study groups from the class to species levels. The most significant differences were *Filobasidiales* family, *Pyronemataceae* family, and *Cryptococcus ater* species, which were enriched in PCa patients compared to controls. The increased *Bipolaris genus* was associated with low prostate-specific antigen (PSA) levels, increased *Sordariomycetes* class was associated with severe pathological stage, and decreased *Phoma herbarum* species was associated with disease relapse, compared to corresponding controls. Several fungi from class to species levels were increased in the controls compared to patients.

**Conclusion:**

This is the first study to show plasma distinct fungal microbiome and its associations with PSA levels, relapse, and pathology stages in PCa patients.

## 1. Background

Prostate cancer (PCa) is one of the most common causes of morbidity and mortality in men. The upper part of the urethra is surrounded by prostate. Retrograde translocation of microorganisms from the urethra to the prostate may lead to chronic bacterial colonization of the prostate [1–4]. Other possible ways of entry of pathogenic organisms into the prostate may be through hematogenous and lymphatic dissemination from the distant locations of infection [5]. Human microbiota affects cancer etiology, progression, and treatment outcomes [6–10]. However, no report has been published on the fungal microbiome in PCa patients *in vivo*. In this study, we report that the plasma fungal microbiome was different in PCa patients compared to controls.

## 2. Methods

### 2.1. Study Subjects

This study was approved by the Institutional Review Board for Human Research at the Medical University of South Carolina (MUSC). Briefly, the plasma samples were deidentified and obtained from the MUSC Biorepository. PSA and pathological grades as well as follow-up clinical data were obtained from existing medical records. PCa relapse was defined to have at least two measurements of blood PSA levels above 0.2 ng/mL after two years of prostatectomy.

### 2.2. Isolation of Microbial DNA from Plasma Samples

Microbial DNA was extracted from 400 *μ*L of plasma and endotoxin-free water control using the QIAamp UCP pathogen Mini kit (catalog number: 50214, Qiagen, Valencia, CA, USA) according to the manufacturer's instructions. We used a DU 640 spectrophotometer (Beckman, city, state) to determine DNA concentration.

### 2.3. Fungal Microbiome Analysis

The detailed method of fungal sequencing was performed using the manufacturer's recommended instructions (http://www.mrdnalab.com). Briefly, forward primer ITS-1 (CTTGGTCATTTAGAGGAAGTAA) and reverse primer ITS-2 (GCTGCGTTCTTCATCGATGC) were used to amplify fungal ITS sequences. The amplicon was sequenced by MR DNA (Shallowater, TX). Sequences were demultiplexed and processed using the UPARSE operational taxonomic unit (OTU) identification pipeline of USEARCH (Edgar) for filtering, removing singletons, and assembling identical sequences. Sequencing data was processed via a proprietary analysis pipeline (MR DNA). Sequences were denoised, and OTUs were defined by clustering at 3% divergence (97% similarity) followed by removal of singleton sequences and chimeras. Final OTUs were taxonomically classified using BLASTn against a database derived from RDPII (http://rdp.cme.msu.edu) and NCBI (http://www.ncbi.nlm.nih.gov). The final OTUs were calculated by the values in plasma samples minus values in the water control.

### 2.4. Statistical Analysis

The OTU table of raw counts was normalized to an OTU table of relative abundances, and taxa of the same type were aggregated at the phylum, class, order, family, and genus levels. Statistical analysis was performed by GraphPad Prism 8.0 (GraphPad, San Diego, USA) using the Mann-Whitney's *U* test (unpaired) and ANOVA. *P* values were adjusted for multiple comparisons by the Benjamini-Hochberg false discovery rate (FDR) or Bonferroni procedure. *P* values of ≤0.05 were considered statistically significant.

## 3. Results

### 3.1. Fungal Diversity of Plasma Fungal Microbiome in PCa Patients and Healthy Control Men

**Figure 1:**
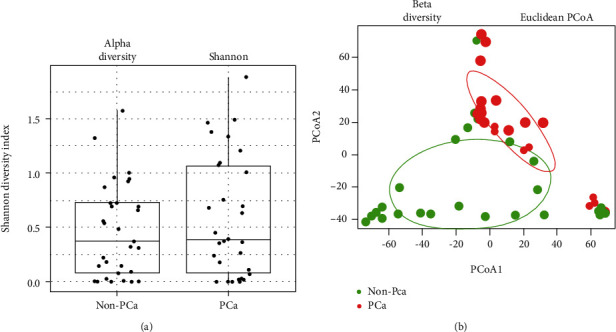
Alpha and beta diversities in prostate cancer patients and healthy men. (a) Alpha diversity and (b) beta diversity of plasma microbiome.

The diversity of plasma fungal microbiome in PCa patients and healthy control men We first analyzed the alpha and beta diversities of the fungal microbiome in plasma and found similar alpha diversity between the two study groups (Figure 1(a)). However, the beta diversity of the circulating fungal microbiome differed in PCa patients and healthy control men (Figure 1(b)). These results imply that the plasma fungal community differs in PCa patients and controls.

### 3.2. Differences in the Relative Abundance of Fungal Classes, Families, Order, and Species in PCa Patients Compared to Controls

**Figure 2:**
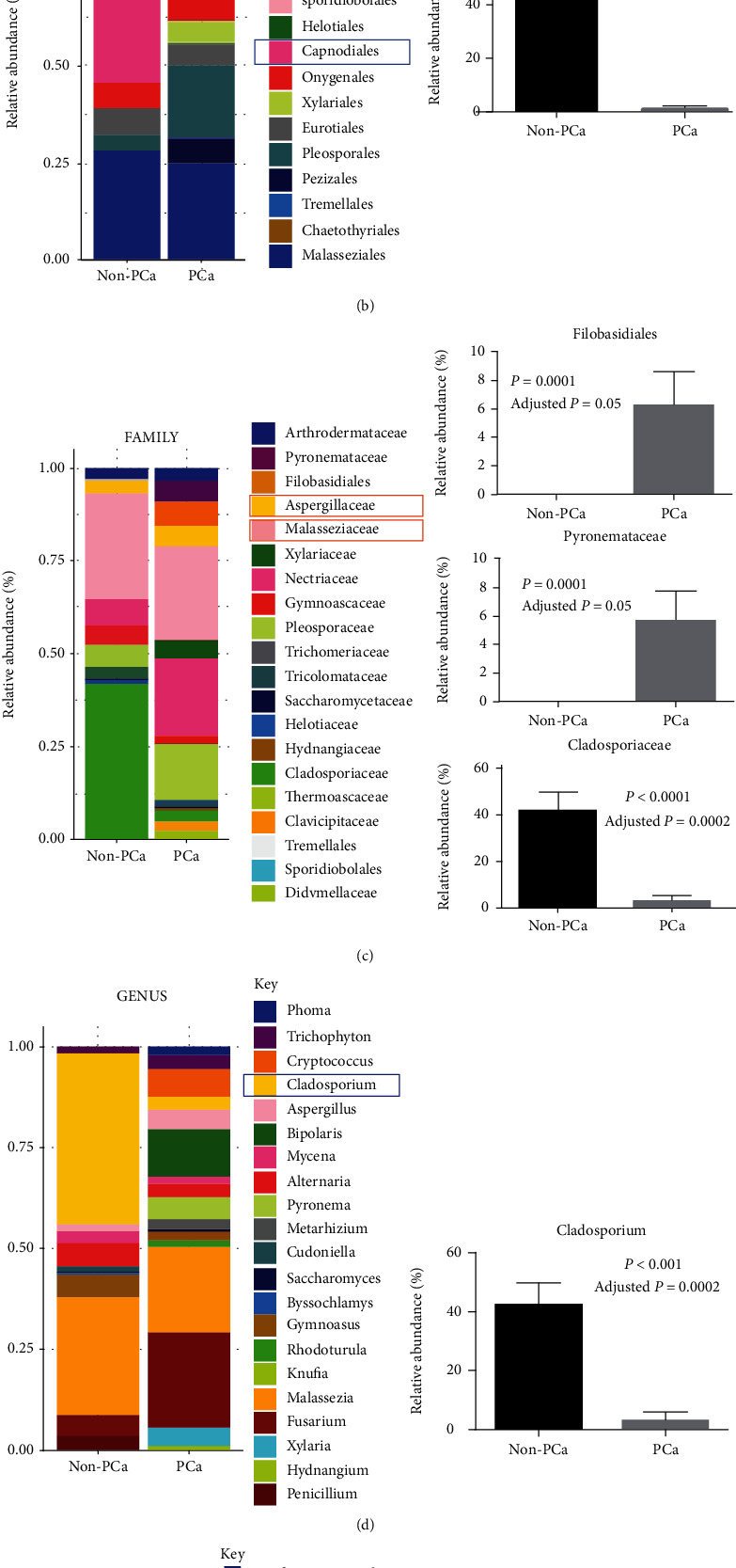
Plasma fungal microbiome in PCa patients and healthy men. (a) In the class level, enrichment of Sordariomycetes was increased in the PCa group (*P* = 0.006) and enrichment of Dothideomycetes (*P* = 0.003) was decreased in PCa patients compared to healthy controls. (b) Order Capnodiales was enriched in healthy controls compared to patients (*P* < 0.0001). (c) Family Filobasidiales and Pyronemataceae (*P* < 0.001) were enriched in PCa patients, and family Cladosporiaceae was reduced in PCa patients compared to controls (*P* < 0.001). (d) Genus Cladosporium was enriched in healthy controls compared to patients (*P* < 0.001). (e) Species *Cryptococcus ater* level was enriched in PCa patients compared to controls (*P* < 0.0001), and species *Cladosporium cladosporioides* was enriched in healthy controls compared to patients (*P* < 0.0001).

At the class level, *Sordariomycetes* was significantly increased in patients with PCa compared to controls (*P* = 0.006, Figure 2(a)). In contrast, class *Dothideomycetes* was significantly decreased in PCa patients compared to controls (*P* = 0.003, Figure 2(a)). At the order level, *Capnodiales* was significantly enriched in the healthy control (*P* < 0.0001, Figure 2(b)). At the family level, *Filobasidiales* and *Pyronemataceae* were significantly increased in PCa patients compared to controls (*P* < 0.001, Figure 2(c)). In contrast, family *Cladosporiaceae* was reduced in PCa patients compared to controls (*P* < 0.001, Figure 2(c)). At the genus level, *Cladosporium* was enriched in the healthy control group compared to patients (*P* < 0.001, Figure 2(d)). At the species level, *Cryptococcus ater* was increased in PCa patients compared to controls (*P* < 0.0001, Figure 2(e)), and *Cladosporium cladosporioides* was decreased in patients compared to healthy controls (*P* < 0.0001, Figure 2(e)).

### 3.3. Circulating Fungal Microbiome and PCa Pathogenesis

**Figure 3:**
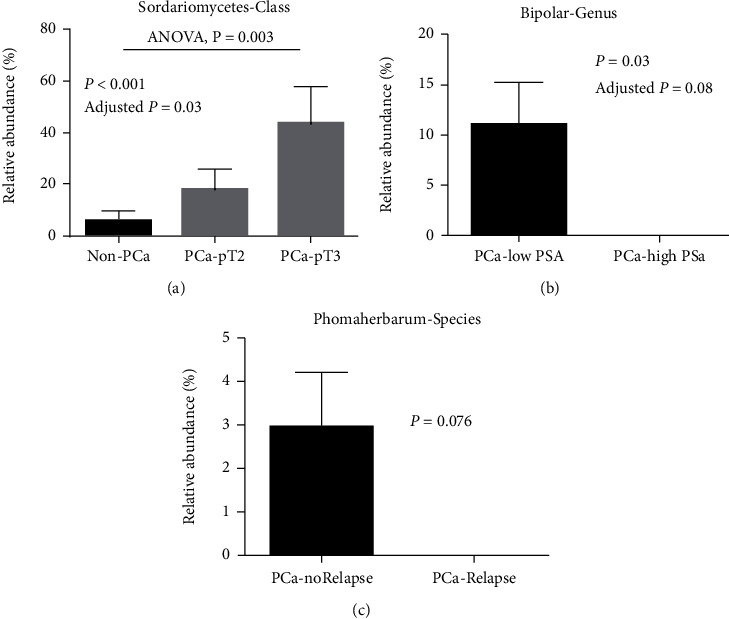
Associations between plasma fungal microbiome and PCa pathogenesis. (a) Increased abundance of class Sordariomycetes was observed in PCa patients with advanced stage tumor (pT3 to pT4) compared to healthy controls. (b) Enriched plasma Bipolaris genus in patients with low level of PSA compared to patients with high level of PSA. Low PSA level was identified as equal or lower than 10 ng/mL, and high PSA was identified as higher than 10 ng/mL. (c) Enriched plasma *Phoma herbarum* species was found in patients without relapse compared to patients with relapse after two years of prostatectomy.

Among PCa patients, the enrichment of class *Sordariomycetes* was significantly increased in patients with advanced pathological grade (pT3 or pT4) compared to patients with pathological grades equal or less than pT2 (*P* = 0.003, Figure 3(a)). The genus *Bipolaris* was enriched in PCa patients with PSA levels lower than 10 ng/mL in plasma compared to patients with PSA levels equal or above 10 ng/mL (*P* = 0.03, Figure 3(b)). The abundance of species *Phoma herbarum* tended to increase in PCa patients without relapse from prostatectomy after two years of following-up compared to patients with relapse, but there was no statistical significance (*P* = 0.076, Figure 3(c)).

## 4. Discussion

Blood and tissues were previously thought sterile unless in the condition of sepsis or infection. However, several recent studies show evidence of a systemic microbiome in the blood and tissues from individuals with cancer as well as other diseases [1–5, 11]. In cancer, microbial communities that occupy specific cancer areas often differ from those at the mucosal sites. Research interests have focused on the role of cancer-associated microbial enrichment in cancer etiology and disease progression [12–16]. Furthermore, infection and inflammation in the prostate are associated with an increased risk of PCa [7], which may result from the infiltration of inflammatory cells to the prostatic and may contribute to the development and progression of PCa [13]. Indeed, microbial toll-like receptor downstream inflammatory cytokines (e.g., IL-6) contribute to proliferative inflammatory atrophy (PIA), which is a precursor of prostatic intraepithelial neoplasia (PIN) and PCa [11, 14].

In this study, we found that class *Sordariomycetes* was significantly increased in patients with PCa compared with controls, as well as in patients with high pathological grade of tumor compared to patients with low pathological grade. However, a previous study shows that the chemical extract of class *Sordariomycetes* has cytotoxicity activity against PC3 cells [13]. We do not know whether the translocated components of *Sordariomycetes* in PCa in vivo were different from those in previous in vitro studies. Moreover, family *Filobasidiales*, family *Pyronemataceae*, and species *Cryptococcus ater* were increased in PCa patients compared to controls. However, there are no relevant studies to analyze the relationship between these fungi and cancer pathology as well as the mechanisms involved. In contrast, the species *Cladosporium cladosporioides* was enriched in the healthy control group compared to patients. In previous studies using McF-7 cells, *cladosporol A* isolated from *Cladosporium cladosporioides* prevented microtubule activation, induced cell apoptosis through ROS-mediated mitochondrial pathway, and upregulated the expression of p21 protein, which contributes to apoptosis and autophagy death of human breast cancer cells [14]. Thus, *Cladosporium cladosporioides* may have a similar mechanism to prevent PCa and reduce disease progression. In another study, *demethoxyfumitremorgin C* isolated from Marine fungus Aspergillus fumigatus induced apoptosis of PC3 cells, one of the human PCa cell lines via caspase cascade response and RAS/Bcl-2-related signaling pathways [15]. These studies and ours may have implications for the effect of fungi or fungal antigen translocation to the circulation on PCa development and progression, but investigations are needed to verify their function.

There are several limitations in this study. First, the samples in this study were only peripheral blood without other key specimens including pathological tissues, feces, urine, or saliva to compare with the results from plasma. Fungi can be found in the gut, oral cavity, and urine [2, 7, 16]; thus, these samples may be important for identifying the origin, sources, or locations and investigating the translocation mechanism of fungi related to PCa. Secondly, the sample size was relatively small and it is a cross-sectional study, which prevent us to draw further conclusions. Nonetheless, this is the first study to show fungal microbiome in plasma from patients with PCa and healthy controls in vivo, which may open a potent important field in PCa in the future.

## 5. Conclusion

This is the first study to show the plasma fungal microbiome in patients with PCa and healthy controls, potentially opening a powerful and important area for future PCa research.

## Data Availability

The datasets generated and/or analyzed during the current study are not publicly available due to privacy or ethical restrictions but are available from the corresponding author on reasonable request.

## Abbreviations

PCa: Prostate cancer
PSA: Prostate specific antigen
MUSC: Medical University of South Carolina.
